# Prediction Model for Risk of Death in Elderly Critically Ill Patients with Kidney Failure

**DOI:** 10.3390/medicina61040640

**Published:** 2025-04-01

**Authors:** Jinping Zeng, Feng Ye, Jiaolan Du, Min Zhang, Jun Yang, Yinyin Wu

**Affiliations:** 1 Department of Epidemiology and Health Statistics, School of Public Health, Hangzhou Normal University, Hangzhou 311121, China; 2 Department of Nutrition and Toxicology, School of Public Health, Hangzhou Normal University, Hangzhou 311121, China

**Keywords:** kidney failure, mortality risk, machine learning, intensive care unit, the elderly

## Abstract

*Background and Objectives*: Kidney failure (KF) is associated with high mortality, especially among critically ill patients in the intensive care unit (ICU). Conversely, age is an independent risk factor for the development of KF. Therefore, understanding the mortality risk profile of elderly critically ill patients with KF can help clinicians in implementing appropriate measures to improve patients’ prognosis. The aim of this study was to construct high-performance mortality risk prediction models for elderly ICU patients with KF using machine learning methods. *Materials and Methods*: Elderly (≥65 years) ICU patients diagnosed with KF were selected and relevant information (including demographic details, vital signs, laboratory tests, etc.) was collected. They were randomly divided into training, validation, and test sets in a 6:2:2 ratio. Logistic regression (LR), random forest (RF), support vector machine (SVM), and extreme gradient boosting (XGBoost) methods were employed to develop prediction models for the risk of death in these elderly KF patients. The model’s performance was evaluated by the receiver operating characteristic curve, precision rate, recall rate, and decision curve analysis. Finally, breakdown plots were utilized to analyze the mortality risk of elderly KF patients. *Results*: A total of 8010 elderly ICU patients with KF were included in this study, among whom 1385 patients died. Mortality prediction models were constructed using various methods, with the areas under the curve (AUC) for the different models being 0.835 (LR model), 0.839 (RF model), 0.784 (SVM model), and 0.851 (XGBoost model), respectively. The integrated Brier score (IBS) for these models were 0.206 (LR model), 0.158 (RF model), 0.217 (SVM model), and 0.102 (XGBoost model), indicating that the XGBoost model and RF model exhibited superior differentiation and calibration capacity. Further analysis revealed that the XGBoost model outperformed the others in terms of both prediction accuracy and stability. Finally, based on the ranking of important features, the primary influencing factors for elderly KF patients were identified as urine output, metastatic solid tumor, body weight, body temperature, and severity score. *Conclusions*: Several high-performing predictive models for mortality risk in elderly ICU patients with KF have been developed using various machine learning algorithms, with the XGBoost model demonstrating the best performance.

## 1. Introduction

Kidney failure (KF) is a disease state defined by the partial or total loss of renal function due to the progression of various chronic kidney diseases to their terminal stage [1]. Age is an independent risk factor for the development of KF, as the structure and function of the kidneys are gradually affected by the increasing number of underlying diseases and medications associated with the aging process [2]. KF is characterized by high morbidity, high mortality, irreversible pathogenesis, expensive treatment, etc. [3]. Therefore, with the growing aging population, KF has emerged as one of the major health- and life-threatening diseases. The key to treating KF patients lies in proactive prevention and intervention, especially for those who are critically ill. Understanding the mortality risk profile of critically ill patients with KF can help identify risk factors associated with KF prognosis, thus guiding clinicians to implement appropriate measures to improve patient outcomes. Consequently, studying the risk of death in elderly patients with KF during the critical care diagnosis and treatment stages is of great significance for improving the overall prevention and treatment of KF.

With the rapid advancement in computer science, the prevalence of electronic medical records is increasing, generating a wealth of usable medical data. Critical care databases are among the most common types of databases, enhancing data availability due to the gradual standardization of clinical data for patients in the intensive care unit (ICU) and providing substantial data support for research on various diseases. Moreover, the development of computer technology and the establishment of medical databases have brought machine learning techniques to the attention of a growing number of researchers. Machine learning is the process of learning the structure of data through numerous datasets and is used to enhance estimation algorithms for specific objectives, and to improve model performance by adjusting parameters [4]. For binary classification problems (such as death/survival), several machine learning methods can be applied for prediction model construction to achieve high performance. For example, logistic regression (LR) is a generalized linear regression analysis model that belongs to supervised learning in machine learning, primarily serving as a classification model to address binary classification problems [5]. The LR model is frequently used in data mining, automated disease diagnosis, and various other fields. Random forest (RF) is another common method in machine learning, essentially an enhanced decision tree algorithm [6]. The RF algorithm is noted for its fast processing of high-dimensional data and its ability to assess the importance of individual features. The extreme gradient boosting (XGBoost) algorithm is a machine learning technique that integrates both linear model solvers and tree learning algorithms [7]. Owing to its fast-computing speed, strong parallelism, scalability, and other attributes, it has gained widespread use in major machine learning competitions in recent years [8]. Support vector machine (SVM) is another machine learning algorithm that also functions as a classification algorithm, aiming to identify a hyperplane that separates different categories. The SVM algorithm is distinguished by its strong generalization ability, enhanced robustness, and interpretability [9]. Therefore, the SVM algorithm is extensively used in biomedical fields [10,11].

The purpose of this study was to construct mortality risk prediction models for elderly critically ill patients with KF using various machine learning methods and to identify the one with the best performance to assist clinicians in predicting and assessing the mortality risk of these patients.

## 2. Methods

### 2.1. Database

Medical information mart for intensive care-IV (MIMIC-IV) is a multi-parameter, structured, single-center database established by the Massachusetts Institute of Technology and Beth Israel Deaconess Medical Center. This database contains detailed clinical information from the ICU at the medical center (https://physionet.org/content/mimiciv/1.0/) (accessed on 13 September 2022) [12]. MIMIC-IV v1.0 was used in this study, with this version released on 16 March 2021.

The MIMIC-IV database contains data on ICU admissions from 2008 to 2018, with patients’ identifying information anonymized. Therefore, obtaining informed consent from the patients was unnecessary. The researchers involved in this study completed all the required training courses and were certified to access the database.

### 2.2. Data Extraction

First, data for KF patients were screened from the MIMIC-IV database. The inclusion criteria included the following: patients diagnosed with KF at the time of admission, aged over 65 years; ICU stay of at least 24 h; and only data from the first admission were selected for patients with multiple admissions. The exclusion criteria were as follows: patients not admitted to the ICU; patients who died within 24 h of admission; and patients with incomplete data. A total of 9269 records and 197 variables were included in this study.

Then, the data were processed using the R software version 4.0.1. The software packages used for the data analysis process include mice, VIM, pROC, dca, xgboost, etc. Demographic information included age, gender, ethnicity, and marital status. The ICU information primarily consisted of the type of the first ICU admission and the length of ICU stay. Vital signs at the first ICU admission included body temperature, heart rate, blood pressure, respiratory rate, and pulse oxygen saturation (SpO2). Laboratory tests at the first ICU admission included hemoglobin, platelets, anion gap, urine output, creatinine, etc. The medications administered during the first ICU admission included norepinephrine, dopamine, vasopressor, dobutamine, phenylephrine, etc. Commonly used critical care scores included sequential organ failure assessment (SOFA), acute physiology score-III (APSIII), logistic organ dysfunction system (LODS), and others. Comorbidities mainly included myocardial infarction, congestive heart failure, mild liver disease, severe liver disease, metastatic solid tumor, etc. Due to the high sampling frequency, the maximum, minimum, and average values were used to represent vital signs and laboratory test results.

### 2.3. Statistical Analysis

Elderly patients with KF were divided into two groups (death/survival) based on 1-year mortality as the end-point. Categorical variables were described using numbers and percentages, and between-group comparisons were conducted with the chi-square test or Fisher’s exact test [13]. For continuous variables, they can be expressed in terms of mean and standard deviation or median and quartile, and differences between the groups can be compared using a T-test or Wilcoxon signed rank test [14]. The dataset was divided into a training set, validation set, and test set in a 6:2:2 ratio. The training set was utilized to develop the mortality risk prediction model, the validation set was employed to refine and optimize the model, and the test set was primarily used to assess or evaluate the model’s performance.

In the model-building stage, backward stepwise regression analysis was first utilized to screen variables with a *p*-value less than 0.05 and combine them with clinical significance to construct the death risk prediction model. The validation set was then utilized to adjust the parameters and maintain the model in an optimal state through the grid search method.

In the model evaluation stage, the differentiation and calibration of the model were first assessed using the receiver operating characteristic curve (ROC) and the integrated Brier score (IBS), and the model’s performance was judged by combining precision and recall. Subsequently, the clinical utility and degree of fit of the models were examined through decision curve analysis (DCA) and residual analysis. Finally, the model with the highest overall diagnostic value was selected, and breakdown plots were created to predict the impact of different variables on outcome indicators.

## 3. Results

### 3.1. Baseline Characteristics

#### 3.1.1. Categorical Variables

In this study, all the categorical variables were expressed using numbers and percentages, and differences between the groups were compared by the chi-square test or Fisher’s exact test. As shown in Table 1, the majority of clinical characteristics in the data from the two groups were significantly different (*p* < 0.05), except for the presence of myocardial infarction, congestive heart failure, dementia, rheumatism, peptic ulcer, diabetes, and AIDS.

#### 3.1.2. Continuous Variables

All the continuous variables were examined for normality, and it was found that none of these variables followed a normal distribution. Therefore, the median and inter-quartile were used for all the continuous variables, and differences between the groups were compared by the Wilcoxon signed rank test. As shown in Table 2, most variables in the two groups were significantly different (*p* < 0.05), with the exception of calcium_max, sodium_min, sodium_max, potassium_min, glucose_max, and temperature_max.

### 3.2. Model Construction

Backward stepwise regression analysis was first employed to filter out variables with a *p*-value less than 0.05 and integrate them with clinical significance to develop the death risk prediction model. LR, RF, SVM, and XGBoost were chosen to construct the mortality risk prediction model. The parameter settings varied for each model, and the validation set was mainly used to fine-tune the parameters and maintain the model in optimal condition through the grid search method. LR model: no additional parameter settings; RF model: the main parameters were mtry = 88 and ntree = 200; and SVM model: the main parameters were C = 10 and gamma = 0.01.

XGBoost model: the main parameters were eta = 0.1 and max_depth = 4.

### 3.3. Model Comparison

#### 3.3.1. Differentiation and Calibration

**Figure 1:**
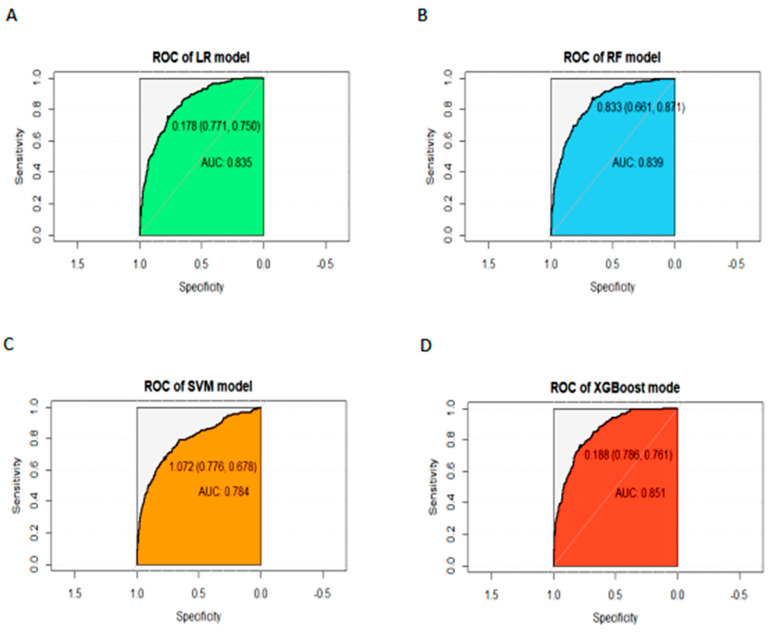
ROC curves for different models: (**A**) LR model, AUC value is 0.835; (**B**) RF model, AUC value is 0.839; (**C**) SVM model, AUC value is 0.784; (**D**) XGBoost model, AUC value is 0.851.

As shown in Table 3 and Figure 1, the area under the curve (AUC) values for the four machine learning models were 0.835 (LR model), 0.839 (RF model), 0.784 (SVM model), and 0.851 (XGBoost model), respectively, with a significant difference among the four models (*p* = 0.011). The IBSs were 0.206 (LR), 0.158 (RF), 0.217 (SVM), and 0.102 (XGBoost), indicating that the RF and XGBoost models performed better in terms of differentiation and calibration. The precision values were 0.730 (LR), 0.805 (RF), 0.794 (SVM), and 0.837 (XGBoost), respectively. The recall values were 0.630 (LR), 0.761 (RF), 0.556 (SVM), and 0.734 (XGBoost), respectively. The precision and recall analysis of these models also revealed that the RF and XGBoost models outperform the other two. Therefore, these two models were selected for further comparison.

#### 3.3.2. Clinical Utility and Degree of Fit

**Figure 2:**
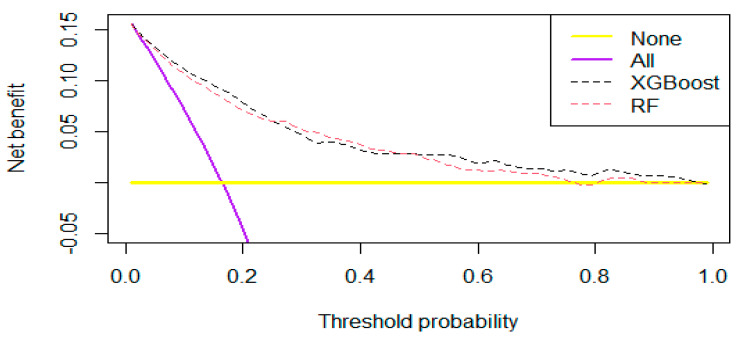
Comparison of the DCA between the RF model and XGBoost model. The *X*-axis indicates the threshold probability for critical care outcome and *Y*-axis indicates the net benefit. The dotted black line = XGBoost model; dotted red line = LR model. The preferred model is the XGBoost model.

**Figure 3:**
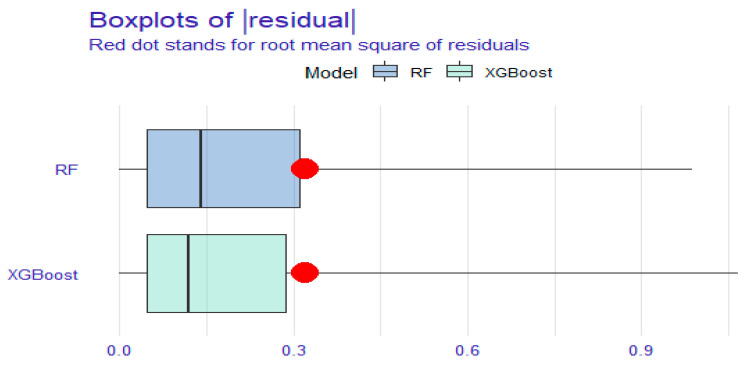
Residual boxplot of RF model and XGBoost model. Red dot stands for root mean square of residuals.

**Figure 4:**
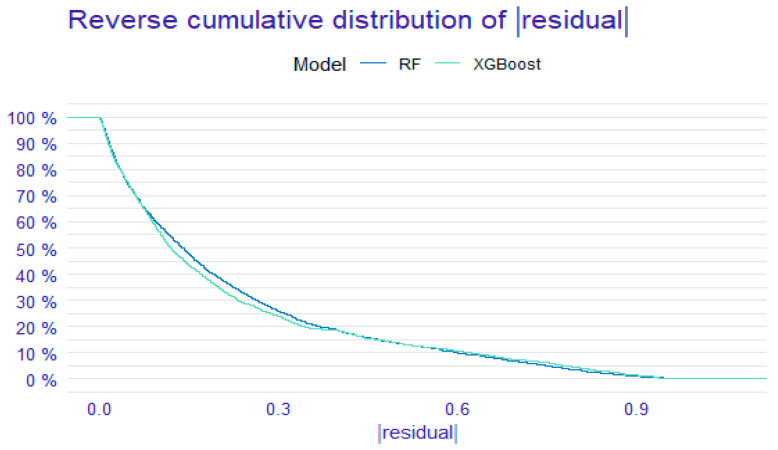
Inverse residual cumulative distribution of two models. The *X*-axis indicates the absolute residual value and the *Y*-axis indicates the cumulative percentage of residuals. Solid blue line = RF model; solid sky blue line = XGBoost model. The preferred model is the XGBoost model.

The DCA and residual analyses of the RF and XGBoost models were compared. The DCA results (Figure 2) indicated that the net benefit of the XGBoost model had a broader range than that of the RF model, suggesting that the XGBoost model possesses greater clinical utility. As shown in Figure 3 and Figure 4, the sample residuals and root mean square residuals of the XGBoost model were relatively small, indicating a superior fitting effect of the XGBoost algorithm and that the model’s predicted values were closer to the actual values. The findings from the DCA and residual analyses of the two models indicated that the XGBoost algorithm model is the more effective model for predicting the risk of death in elderly KF patients.

**Figure 5:**
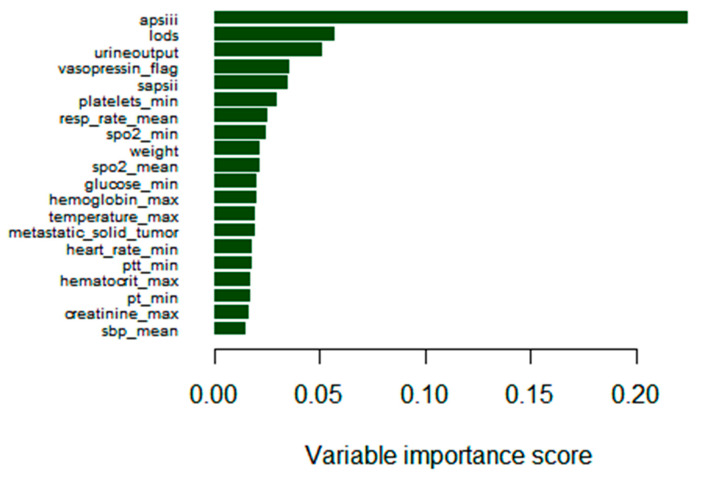
The top 20 important features in the XGBoost model.

To summarize, the XGBoost model demonstrated the best performance. The higher AUC value of the XGBoost model indicates superior differentiation ability. Additionally, the small IBS value suggests that the XGBoost model is well calibrated. The precision and recall of the XGBoost model were both relatively high, further indicating its strong performance. Regarding clinical utility, the broader net benefit range of the XGBoost model signifies better clinical utility. Moreover, the degree of fit of the XGBoost model was superior, as evidenced by the smaller sample residuals and root mean square residuals. Subsequently, the XGBoost model was employed to select the top 20 features based on importance to create a breakdown plot (Figure 5).

### 3.4. Breakdown Plot for the XGBoost Model

**Figure 6:**
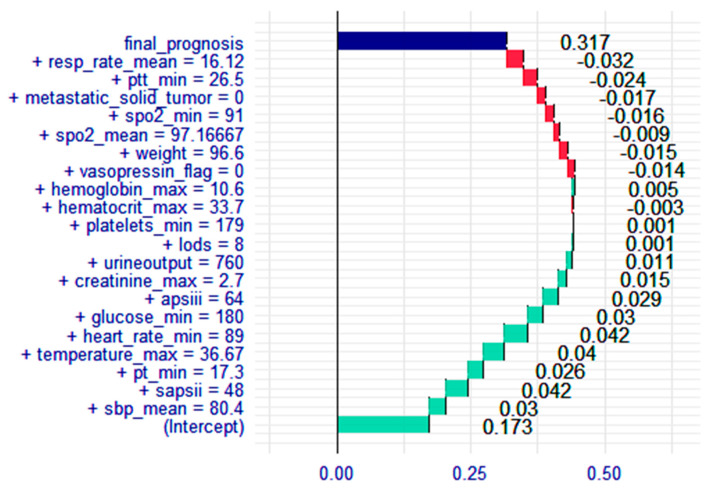
Breakdown plot of the XGBoost model. The plot sorts features by their distribution to the prediction. The color represents the impact on the model output (red negative, green positive). The top of the plot shows the final prediction output of the model.

Through the analysis of multidimensional model performance assessment indexes, it was determined that the XGBoost model demonstrated the best performance. Consequently, breakdown plots were constructed based on XGBoost to predict the risk of death in elderly patients with KF. In Figure 6, the extent of each indicator’s contribution to the outcome variables can be clearly observed. These variables included urine output, vasopressor, metastatic solid tumor, body weight, body temperature, and severity score, all of which were closely associated with a high risk of death in elderly patients with KF.

## 4. Discussion

KF is a prevalent disease characterized by high morbidity, mortality, poor prognosis, and expensive treatment costs. It has become a major health concern affecting global life and well-being. KF is most frequently observed in the ICU, where it presents a high mortality rate, is challenging to prevent, and creates numerous difficulties for clinicians. An epidemiological survey revealed that ICU patients are more likely to develop KF, with the incidence of KF in critically ill patients being considerably higher than in other general hospitalized patients [15]. Another study focusing on ICU patients showed that the incidence of KF reached as high as 50%, with most patients developing KF within a few days of their ICU admission [16].

In contrast to those earlier studies that used the MIMIC database to predict KF in the general ICU population [17,18], the present study focused on elderly ICU patients to examine the risk of death from KF. This emphasis is due to the fact that age-related changes in kidney structure and function render elderly ICU patients more vulnerable to KF [19]. In our study, four machine learning methods, i.e., LR, RF, SVM, and XGBoost, were used to establish prediction models for the risk of death in elderly KF patients using the MIMIC-IV database. Among all the models, the SVM model performs relatively poorly. When the number of samples is large, the storage and calculation of the SVM model will consume a lot of machine memory and operation time [20]. Therefore, the SVM model has difficulty obtaining better results in this kind of research. Among these, the XGBoost model demonstrated the best performance in predicting the risk of death from KF in the elderly. XGBoost is an efficient and flexible machine learning algorithm that can improve the model performance by increasing the learning rate and maximum tree depth [21]. The research results indicated that the XGBoost model achieved higher accuracy compared to the other machine learning methods, in particular when dealing with high dimensional data. Similarly, Yue et al. [22] reported that the XGBoost model was the most effective among all the prediction models when assessing the risk of KF in patients with sepsis. Deng et al. [23] also found that the XGBoost algorithm outperformed other machine learning models in predicting KF prognosis in hospitalized children. In addition, a meta-analysis concluded that XGBoost was more effective than other machine learning algorithms in predicting KF [24]. These results collectively suggest that the XGBoost model surpasses many other types of machine learning methods.

In addition, breakdown plots were constructed for interpreting the optimal predictive model, focusing on analyzing the extent to which each indicator contributes to the outcome variable. Breakdown plots also serve as a visualization tool to assess the impact of the specific values of each variable on the model’s prognosis. They assist physicians in formulating the best medical treatment plan for patients and provide reliable conclusions for the study. A patient was randomly selected for analysis, and breakdown plots clearly demonstrated that indicators with varying values had different impacts on the patient. For example, decreased urine output is related to a higher probability of KF, and as shown in Figure 6, it also positively influenced the predicted outcome, increasing the risk of death in this patient. The breakdown plots improved the transparency and interpretability of machine learning models, making it easier for the public to understand the inner workings of the predictive models.

By using the breakdown plots to assess the significance of the various model features, it became possible to identify the major factors associated with the high risk of death in elderly KF patients, including urine output, vasopressor use, metastatic solid tumor, body weight, body temperature, and severity score. Firstly, among the critical care scores, APSIII stands out as the most significant predictor of mortality risk in elderly patients with KF. Meng et al. [25] reported that APSIII is a commonly used tool for determining disease severity and predicting mortality risk, demonstrating effectiveness in the timely identification of high-risk patients and the formulation of intervention strategies. Secondly, among the identified physiologic indicators, urine output has long been recognized as a key factor in KF. Urine output often emerges as the initial clinical sign of KF, and reduced urine output, which can lead to hypovolemia, heightens the risk of death in KF patients [26]. Prompt rehydration therapy restores circulating blood volume and improves impaired renal perfusion [27]. Body temperature is an easily measurable indicator, and when it drops below average, it may result in decreased renal blood flow, potentially leading to impaired tubular function [28]. Indeed, KF occurs in approximately half of patients with hypothermia in the ICU. Body weight is also a significant factor influencing mortality in elderly KF. It has been shown that the risk of death in elderly patients with KF is directly proportional to body weight [29]. Overweight individuals are typically associated with high blood pressure and other metabolic abnormalities, which also increase the risk of acidosis, eventually increasing the risk of death in elderly patients with KF. Lastly, the presence of certain medications or comorbidities can further influence the risk of death from KF. Vasoactive substances, such as vasopressors, increase glomerular perfusion pressure and urine output, thereby affecting the incidence of KF [30]. On the other hand, metastatic solid tumors are a prevalent comorbidity in elderly patients with KF [31]. Liver disease further elevates the risk of death in patients with KF due to insufficient renal blood perfusion and impaired renal function resulting from significant fluid accumulating in the patient’s abdominal cavity [32]. As these indicators are readily assessed at the time of admission, they can serve as predictors of mortality risk in elderly patients with KF.

This study used breakdown plots to predict the impact of variables on mortality outcomes in the optimal model to improve the transparency and interpretability of machine learning models. There were three main shortcomings of this study: First, the occurrence of KF in elderly patients may be influenced by genetic factors. Therefore, subsequent studies should consider the role of the patients’ genetic background. Second, the MIMIC-IV database did not provide patient history and long-term follow-up events, which may result in the oversight of some key variables. Finally, the data used in this study were sourced solely from the MIMIC-IV database, which lacks external validation. Therefore, these issues should be carefully addressed in future studies. In future studies, we plan to use the eICU Collaborative Research Database to conduct external validation of the prediction model of mortality risk from severe renal failure in the elderly to further refine the content of this study [33].

## 5. Conclusions

In this study, different machine learning methods were employed to develop a mortality risk prediction model for critically ill elderly patients with KF, with the XGBoost model emerging as the most effective. The risk factors of death in elderly KF patients were identified through breakdown plots, which include urine output, vasopressor use, metastatic solid tumors, body weight, body temperature, and severity score.

## Figures and Tables

**Table 1:** Baseline characteristics of patients (categorical variable).

	Death	Survival	*p*
Sample size	1385	6625	
Gender (%)			0.02
Female	643 (46.43)	2842 (42.90)	
Male	742 (53.57)	3783 (57.10)	
Ethnicity (%)			0.003
Caucasian	994 (71.77)	4682 (70.67)	
African American	115 (8.30)	725 (10.94)	
Asian	41 (2.96)	188 (2.84)	
Other	235 (16.97)	1030 (15.55)	
Marital status (%)			<0.001
Single	245 (17.69)	1179 (17.80)	
Married	632 (45.63)	3102 (46.82)	
Other	508 (36.68)	2344 (35.38)	
First care unit (%)			<0.001
MICU	350 (25.27)	1631 (24.62)	
SICU	320 (23.10)	1237 (18.67)	
Other	715 (51.63)	3757 (56.71)	
Dialysis (%)			<0.001
Yes	67 (4.84)	131 (1.98)	
No	1318 (95.16)	6494 (98.02)	
Antibiotic (%)			<0.001
Yes	1314 (94.87)	5694 (85.95)	
No	71 (5.13)	931 (14.05)	
Dobutamine (%)			<0.001
Yes	100 (7.22)	159 (2.40)	
No	1285 (92.78)	6466 (97.60)	
Dopamine (%)			<0.001
Yes	126 (9.10)	300 (4.53)	
No	1259 (90.90)	6325 (95.47)	
Nerve blockers (%)			<0.001
Yes	80 (5.78)	91 (1.37)	
No	1305 (94.22)	6534 (98.63)	
Epinephrine (%)			<0.001
Yes	141 (10.18)	358 (5.40)	
No	1244 (89.82)	6267 (94.60)	
Norepinephrine (%)			<0.001
Yes	738 (53.29)	1780 (26.87)	
No	647 (46.71)	4845 (73.13)	
Phenylephrine (%)			<0.001
Yes	425 (30.69)	1182 (17.84)	
No	960 (69.31)	5443 (82.16)	
Vasopressor (%)			<0.001
Yes	392 (28.30)	465 (7.02)	
No	993 (71.70)	6160 (92.98)	
Myocardial infarct (%)			0.32
Yes	398 (28.74)	1816 (27.41)	
No	987 (71.26)	4809 (72.59)	
Congestive heart failure (%)			0.05
Yes	749 (54.08)	3392 (51.20)	
No	636 (45.92)	3233 (48.80)	
Peripheral vascular disease (%)			0.03
Yes	256 (18.52)	1067 (16.11)	
No	1129 (81.48)	5558 (83.89)	
Cerebrovascular disease (%)			<0.001
Yes	240 (17.33)	896 (13.52)	
No	1145 (82.67)	5729 (86.48)	
Dementia (%)			0.90
Yes	109 (7.87)	528 (7.97)	
No	1276 (92.13)	6097 (92.03)	
Chronic pulmonary disease (%)			0.04
Yes	473 (34.15)	2077 (31.35)	
No	912 (65.85)	4548 (68.65)	
Rheumatic disease (%)			0.89
Yes	65 (4.69)	305 (4.60)	
No	1320 (95.31)	6320 (95.40)	
Peptic ulcer disease (%)			0.26
Yes	65 (4.69)	267 (4.03)	
No	1320 (95.31)	6358 (95.97)	
Diabetes complicated (%)			0.15
Yes	395 (28.52)	2020 (30.49)	
No	990 (71.48)	4605 (69.51)	
Mild liver disease (%)			<0.001
Yes	253 (18.27)	602 (9.09)	
No	1132 (81.73)	6023 (90.91)	
Paraplegia (%)			0.005
Yes	69 (4.98)	227 (3.43)	
No	1316 (95.02)	6398 (96.57)	
Malignant cancer (%)			<0.001
Yes	368 (26.57)	1024 (15.46)	
No	1017 (73.43)	5601 (84.54)	
Severe liver disease (%)			<0.001
Yes	129 (9.31)	236 (3.56)	
No	1256 (90.69)	6389 (96.44)	
Metastatic solid tumor (%)			<0.001
Yes	205 (14.80)	391 (5.90)	
No	1180 (85.20)	6234 (94.10)	
Aids (%)			0.48
Yes	3 (0.21)	9 (0.14)	
No	1382 (99.78)	6616 (99.86)	

SICU—surgical intensive care unit; MICU—medical intensive care unit.

**Table 2:** Baseline characteristics of patients (continuous variable).

	Death	Survival	*p*
Sample size	1385	6625	
Age, year	79.40 (72.66, 86.48)	78.13 (71.46, 84.91)	<0.001
Weight, kg	74.80 (63.50, 87.90)	77.90 (66.30, 91.47)	<0.001
Length of stay in the ICU, day	5.09 (2.50, 10.27)	3.09 (1.91, 6.01)	<0.001
Hematocrit_min (%)	27.40 (23.70, 32.20)	28.40 (24.50, 33.00)	<0.001
Hematocrit_max (%)	31.70 (28.00, 35.90)	32.50 (29.10, 36.90)	<0.001
Hemoglobin_min (g/dL)	8.80 (7.60, 10.50)	9.30 (8.00, 10.80)	<0.001
Hemoglobin_max (g/dL)	10.20 (8.90, 11.60)	10.60 (9.40, 12.10)	<0.001
Platelets_min (k/uL)	165.00 (100.00, 226.00)	167.00 (121.00, 224.00)	<0.001
Platelets_max (k/uL)	199.00 (132.00, 266.00)	201.00 (153.00, 266.00)	<0.001
WBC_min (k/uL)	9.80 (7.00, 13.50)	9.50 (6.90, 12.50)	<0.001
WBC_max (k/uL)	12.9 (10.00, 18.00)	12.70 (9.20, 17.10)	<0.001
AG_min (mEq/L)	14.00 (12.00, 17.00)	13.00 (11.00, 15.00)	<0.001
AG_max (mEq/L)	17.00 (15.00, 21.00)	17.00 (14.00, 19.00)	<0.001
Bicarbonate_min (mEq/L)	20.00 (16.00, 23.00)	21.00 (18.00, 24.00)	<0.001
Bicarbonate_max (mEq/L)	23.00 (20.00, 26.00)	23.00 (21.00, 26.00)	<0.001
BUN_min (mg/dL)	33.00 (24.00, 50.00)	30.00 (21.00, 42.00)	<0.001
BUN_max (mg/dL)	39.00 (29.00, 58.00)	36.00 (25.00, 50.00)	<0.001
Calcium_min (EU/dL)	8.00 (7.50, 8.50)	8.10 (7.70, 8.60)	<0.001
Calcium_max (EU/dL)	8.60 (8.00, 9.00)	8.60 (8.10, 9.00)	0.09
Chloride_min (mEq/L)	101.00 (97.00, 106.00)	102.00 (98.00, 106.00)	<0.001
Chloride_max (mEq/L)	105.00 (101.00, 109.00)	106.00 (102.00, 109.00)	<0.001
Creatinine_min (g/dL)	1.30 (1.10, 2.00)	1.30 (1.00, 1.70)	<0.001
Creatinine_max (g/dL)	1.60 (1.30, 2.30)	1.60 (1.30, 2.10)	<0.001
Sodium_min (mEq/L)	137.00 (134.00, 140.00)	137.00 (134.00, 140.00)	0.05
Sodium_max (mEq/L)	140.00 (137.00, 143.00)	140.00 (137.00, 143.00)	0.30
Potassium_min (mEq/L)	4.00 (3.60, 4.50)	4.00 (3.60, 4.40)	0.33
Potassium_max (mEq/L)	4.60 (4.20, 5.20)	4.60 (4.10, 5.10)	0.04
PT_min (s)	13.70 (13.10, 16.20)	13.50 (12.30, 14.80)	<0.001
PT_max (s)	14.90 (14.10, 18.30)	14.60 (13.00, 16.50)	<0.001
PTT_min (s)	29.30 (27.30, 34.70)	29.20 (26.30, 32.60)	<0.001
PTT_max (s)	32.70 (30.60, 43.80)	32.70 (28.60, 38.00)	<0.001
Glucose_min (mg/dL)	105.00 (87.00, 131.00)	104.50 (88.00, 124.00)	0.02
Glucose_max (mg/dL)	168.00 (135.00, 217.00)	168.00 (133.00, 211.00)	0.14
Urine output (ml)	850.00 (405.00, 1495.00)	1378.00 (863.00, 2120.00)	<0.001
Heart rate_min (min^−1^)	73.00 (63.00, 86.00)	69.00 (60.00, 78.00)	<0.001
Heart rate_max (min^−1^)	107.00 (94.00, 124.00)	99.00 (87.00, 114.00)	<0.001
Heart rate_mean (min^−1^)	89.22 (77.26, 101.69)	81.93 (72.32, 92.81)	<0.001
SBP_min (mmHg)	84.00 (75.00, 92.00)	88.00 (80.00, 98.00)	<0.001
SBP_max (mmHg)	141.00 (126.00, 156.00)	144.00 (132.00, 160.00)	<0.001
SBP_mean (mmHg)	108.07 (100.52, 117.83)	113.53 (105.52, 125.10)	<0.001
DBP_min (mmHg)	41.00 (34.00, 47.00)	43.00 (37.00, 49.00)	<0.001
DBP_max (mmHg)	84.00 (72.00, 97.00)	84.00 (72.00, 97.00)	0.35
DBP_mean (mmHg)	57.80 (51.72, 64.28)	58.50 (52.48, 65.63)	<0.001
Respiratory rate_min (min^−1^)	13.00 (11.00, 16.00)	13.00 (11.00, 15.00)	<0.001
Respiratory rate_max (min^−1^)	30.00 (26.00, 34.00)	28.00 (24.00, 32.00)	<0.001
Respiratory rate_mean (min^−1^)	20.44 (18.04, 23.61)	19.24 (17.13, 21.80)	<0.001
Body temperature_min (°C)	36.39 (36.06, 36.56)	36.39 (36.17, 36.61)	<0.001
Body temperature_max (°C)	37.11 (36.83, 37.50)	37.11 (36.89, 37.44)	0.13
Body temperature_mean (°C)	36.72 (36.47, 36.95)	36.74 (36.54, 36.97)	<0.001
SpO2_min (%)	91.00 (87.00, 94.00)	92.00 (89.00, 94.00)	<0.001
SpO2_max (%)	100.00 (100.00, 100.00)	100.00 (99.00, 100.00)	0.04
SpO2_mean (%)	96.77 (95.08, 98.33)	96.90 (95.50, 98.24)	0.01
SOFA	8.00 (6.00, 12.00)	5.00 (4.00, 8.00)	<0.001
APSIII	75.00 (59.00, 97.00)	50.00 (41.00, 64.00)	<0.001
LODS	9.00 (6.00, 11.00)	5.00 (4.00, 7.00)	<0.001
OASIS	40.00 (34.00, 47.00)	33.00 (28.00, 39.00)	<0.001
SAPSII	52.00 (43.00, 62.00)	41.00 (35.00, 49.00)	<0.001
SIRS	3.00 (2.00, 3.00)	3.00 (2.00, 3.00)	<0.001

WBC—white blood cells; AG—anion gap; BUN—blood urea nitrogen; PT—prothrombin time; PTT—partial thromboplastin time; SBP—systolic blood pressure; DBP—diastolic blood pressure; SpO2—pulse oxygen saturation; SOFA—sequential organ failure assessment; APSIII—acute physiology and chronic health score III; LODS—logistic organ dysfunction system; OASIS—Oxford acute severity of illness score; SAPSII—simplified acute physiology score II; SIRS—systemic inflammatory response syndrome; Max—maximum; Min—minimum.

**Table 3:** Evaluation metrics for machine learning models.

Mode	AUC	Brier	Precision	Recall
LR	0.835	0.206	0.730	0.631
RF	0.839	0.158	0.805	0.761
SVM	0.784	0.217	0.794	0.556
XGBoost	0.851	0.102	0.837	0.734

## Data Availability

The data that support the findings of this study are openly available on the MIMIC-IV website at https://physionet.org/content/mimiciv/1.0/. The datasets used and/or analyzed during the current study are available from the corresponding author upon reasonable request.

## Abbreviations

KF: kidney failure; ICU: intensive care unit; LR: logistic regression; RF: random forest; SVM: support vector machine; XGBoost: extreme gradient boosting; MIMIC-IV: medical information mart for intensive care-IV; APSIII: acute physiology score-III; LODS: logistic organ dysfunction system; ROC: receiver operating characteristic curve; IBS: integrated brier score; DCA: decision curve analysis; AUC: area under the curve; SICU: surgical intensive care unit; MICU: medical intensive care unit; WBC: white blood cells; AG: anion gap; PT: prothrombin time; PTT: partial thromboplastin time; BUN: blood urea nitrogen; SBP: systolic blood pressure; DBP: diastolic blood pressure; SpO2: pulse oxygen saturation; SOFA: sequential organ failure assessment; OASIS: Oxford acute severity of illness score; SPASII: simplified acute physiology score-II; SIRS: systemic inflammatory response syndrome; Max: maximum; Min: minimum.
